# Sensor4PRI: A Sensor Platform for the Protection of Railway Infrastructures

**DOI:** 10.3390/s150304996

**Published:** 2015-02-27

**Authors:** Eduardo Cañete, Jaime Chen, Manuel Díaz, Luis Llopis, Bartolomé Rubio

**Affiliations:** Department of Languages and Computer Science, University of Málaga, Boulevar Louis Pasteur 35, 29071 Málaga, Spain; E-Mails: hfc@lcc.uma.es (J.C.); mdr@lcc.uma.es (M.D.); luisll@lcc.uma.es (L.L.); tolo@lcc.uma.es (B.R.)

**Keywords:** wireless sensor networks, railway infrastructure protection, sensor platform, slab track

## Abstract

Wireless Sensor Networks constitute pervasive and distributed computing systems and are potentially one of the most important technologies of this century. They have been specifically identified as a good candidate to become an integral part of the protection of critical infrastructures. In this paper we focus on railway infrastructure protection and we present the details of a sensor platform designed to be integrated into a slab track system in order to carry out both installation and maintenance monitoring activities. In the installation phase, the platform helps operators to install the slab tracks in the right position. In the maintenance phase, the platform collects information about the structural health and behavior of the infrastructure when a train travels along it and relays the readings to a base station. The base station uses trains as data mules to upload the information to the internet. The use of a train as a data mule is especially suitable for collecting information from remote or inaccessible places which do not have a direct connection to the internet and require less network infrastructure. The overall aim of the system is to deploy a permanent economically viable monitoring system to improve the safety of railway infrastructures.

## 1. Introduction

Wireless Sensor Networks (WSNs) are potentially one of the most important technologies of this century [1]. Due to a combination of advances in electronics, nanotechnology, wireless communications, computing, networking, and robotics, it is now possible to design advanced sensors and sensor systems that can be used in various application areas: environmental monitoring; object and event detection; military surveillance; precision agriculture; transportation, *etc*. [2]. WSNs constitute a pervasive and distributed computing technology with a wide range of applications. A WSN consists of (potentially) thousands of tiny, low-cost and low-power nodes, colloquially referred to as “motes”, which can sense the environment through sensors. The motes get information about some physical parameters from the environment and wirelessly send it to one or several base stations, where it can be analyzed. Wireless sensor networks act as the senses of a huge nervous system that allows real information to be felt, stored and analyzed in an ICT system.

Recent advances in these kinds of systems, with a strong focus on the achievement of strict Quality of Service (QoS) requirements, make WSNs a promising technology for the Critical Infrastructure Protection (CIP) field [3]. Infrastructures such as power distribution and generation, nuclear power plants, railways, water distribution and management, etc. can take advantage of WSN deployments. Proof of this is that the U.S. Department for Homeland Security stated, in the 2004 National Plan for Research and Development in Support for CIP, that one of the strategic goals was “to provide a National Common Operating Picture (COP)” for Critical Infrastructures, where the core of the systems would be an intelligent, self-monitoring, and self-healing sensor network. The Australian government, by means of the Cooperative Research Center for Security (CRC-SAFE), is examining and developing solutions to security problems in CIP systems, including WSNs [4]. Finally, European projects related to CIP have grown considerably in the last five years [5,6].

A good example of a critical infrastructure where WSNs can be applied are railways. As any other kind of infrastructure, they are affected by the aging process. For example, large sections of the railway lines in the United States were built in the late 19th century or beginning of the 20th century. In Europe large sections of the railway lines were rebuilt after the Second World War. Therefore, it is really important to regulate maintenance and restoration guidelines to ensure safety in railway transport. In this regard, more attention has been paid to this issue from the late 20th century onwards. In Europe, the guidelines for the maintenance of railway lines are regulated in documents 96/48/CE and 2001/16/CE. Following these guidelines, in Spain, for example, a visual inspection of the elements of the infrastructure needs to be carried out every 15 years by specialized technicians. Furthermore, a general visual inspection is completed each year by non-specialized railway line guards. This may be sufficient for most railway elements, but in structures with unusual topology or particularly high/long bridges, the information on the evolution of defects is more limited. Moreover the visual inspections are much more difficult to carry out and require a temporary closure to traffic. In these structures, it is common to check the state of the structure using specialized equipment or even install a permanent monitoring system, e.g., fiber optic instrumentation with Brillouin Optical Time Domain Analysis (BOTDA) or distributed sensor instrumentation. One of the main disadvantages of these systems is their high cost, which means that they can only be installed at a few sites. Moreover, if there is no mobile coverage then data acquired by the system cannot be sent to the remote control center. 

The current WSN technology can be used as a permanent monitoring system and considerably reduce the cost of installation and maintenance since no wiring is required. However, a permanent sensor platform is expected to have a long life span, especially if economic viability is taken into account. In this sense, it is very important to analyze the characteristics of the components of the WSN because its behavior will determine the response of the whole system. This paper presents our experiences in the process to select a sensor platform which meets the system requirements. Efficient energy consumption sensors, efficient communication and energy harvesting systems have been studied to guarantee the requirements in both the slab installation phase and the maintenance monitoring stage. In addition, we also believe our approach can cope with the network coverage problem and tackle the transfer of large quantities of data reliably. This second advantage with respect to the aforementioned wired systems, has already been highlighted in one of our previous papers [7]. That work centered on describing how sensor nodes are deployed along the infrastructure, forming clusters, taking periodical readings about the structural health and sending the information to trains passing through. The application scenario was simulated using Cooja [8]. Now, in the present paper we focus on the selection and implementation of the real sensor platform, describing, in more depth, the different sensors and energy devices used, showing several results obtained from the multiple field evaluations we have carried out.

The sensor platform presented in this paper can be applied to any kind of railway infrastructure, but the prototype we have developed is especially tailored to be used in a slab track system. Slab tracks are currently being studied as substitutes for traditional ballasted tracks for railways due to their advantages such a high stability, no maintenance and long life cycles, but at the expense of a higher price. Our sensor platform is designed to be part of the slab tack, being inserted inside it during its construction, so that it can be used in both installation and maintenance phases. The slab track becomes an active element capable of monitoring and reporting information about the environment such as vibration performance, distance and inclination and also assisting operators in the installation phase.

The experiences described in this paper are part of the project entitled Fastrack [9] funded by the Spanish Government’s FEDER program. The main objective is the design of a new slab track system for high speed trains (faster than 250 km/h) that is environmentally and economically sustainable. In this context, a real time monitoring of the system is important to meet the project requirements. The project is currently testing the system in laboratories of the Spanish government institution for the study and experimentation of public works, called CEDEX.

Other technical objectives are:

- Address an affordable and environmentally sustainable manufacturing.
- Have elements that reduce the production of noise and vibrations from railway traffic.
- Low maintenance, increasing the hours of availability of the infrastructure operation.
- Quick and easy repair if necessary, avoiding long route cuts

The rest of the paper is organized as follows: in Section 2 related work is presented. Section 3 presents a description of the system requirements. The platform, its implementation and evaluation are described in Section 4. In Section 5 the evaluation of different energy harvesting mechanisms is presented. Section 6 shows the cost analysis of the Sensor4PRI platform. Finally, Section 7 concludes the paper.

## 2. Related Work 

We can find many proposals that make use of WSNs for railway infrastructure protection in the literature. Topics addressed in these proposals include monitoring the state of the rail infrastructure and trains, detecting obstacles or signaling. Some of these proposals are detailed below.

The details of a real deployment of a WSN on a railway bridge are presented in [10]. The WSN has a total of eight nodes and a TmoteSky base station gathering data on the status of the bridge to detect deformations in the infrastructure when trains cross it. Accelerometer sensors are used to detect trains approaching the bridge and start the process of collecting data that is active while the train is crossing the bridge. The network is automatically organized according to a protocol based on routing trees to be able to transmit information to the base station. Once the information has been received, the UMTS technology is used to send data to a remote control center. 

In [11], another TmoteSky WSN formed to collect information on the status of a railway bridge is presented. A sensor placed on trains is used as a mobile base that collects data from the sensor network as it travels through the bridge station. A tree-based routing protocol is used to transmit information to a number of leader nodes. They transmit the information collected to mobile nodes located on the train. The sampling frequency in [11] (around 20 Hz) is much lower than the one presented in this paper because the train speed is assumed to be low.

A WSN architecture is presented in [12] for monitoring the state of the railways. The sensors make use of accelerometers and ultrasonic sensors to detect wear and tear on roads. A hierarchical network topology is used so that there are multiple paths that can be used to reach the base station. This tolerance is achieved against failure nodes. The data collected by the network are merged as they are sent to the base station through the use of fuzzy logic techniques.

Similar to the one presented in the previous paragraph is the system described in [13]. This also makes use of a hierarchical network and ultrasonics to detect possible problems in the railway’s sensors. It also introduces the use of image processing and the use of electromagnetic detection of dangerous objects on the railway tracks.

Another architecture for the monitoring of railway infrastructure, which includes a WSN as an integral part of it is given in [14]. The infrastructure, called SENSORAIL, integrates different kinds of sensors (such as temperature sensors, cameras, *etc*.) for the protection of such infrastructures. The framework provides information by means of abstractions of high-level programming. It also incorporates a threat detection system based on the information acquired by the sensors. This work focuses on the system architecture instead of the hardware details.

In [15] the installation of permanent sensors for monitoring the condition of train bearings and detecting potential problems in them, such as locked brakes, overheating in bearings, *etc*. is proposed. Two particular aspects are studied: the behavior of the wireless communication system of sensors with respect to where the sensors are installed on the train and energy harvesting techniques to minimize maintenance of the sensors. The results indicate that the radio transmitters perform better when placed above the train than when placed beneath. It also identifies obtaining energy through vibrations as most promising for such applications. The integration of sensors in the wheels makes the energy harvesting easier. In our proposal, vibrations are attenuated due to the integration of nodes inside the slab track.

In [16] WSN technology is used to monitor the integrity of freight trains. Specifically, sensors are used to detect situations in which wagons become separated from the locomotive for unjustified reasons. Accelerometer and vibration sensors are able to detect whether the train is moving. They propose mechanisms for energy harvesting. However, the hardware prototype does not include any of these proposals.

In [17] the use of WSN is proposed for early earthquake detection and control of security in railway networks. The WSN deployed in areas with high seismic activity is used to detect the onset of an earthquake with enough time to communicate the information to the control center. The latter, depending on the location of the earthquake and its severity, would take the most appropriate decision, for example completely stop the train.

In [18] a WSN based on Zigbee is proposed for monitoring railway tracks. Specifically, distance sensors and vibration are used to report the state of the route and the presence of other trains. They propose piezoelectric sensors to detect vibrations and do not study any energy harvesting mechanism.

In general, the majority of the proposals present a WSN architecture to monitor the infrastructure (railways, bridges, *etc*.) but they do not detail the different situations which led them to select the components of the hardware architecture. For example, the accelerometer sensor selection will depend on the sampling frequency and the train speed. Also, the piezoelectric sensors typically used for energy harvesting will depend on the vibration range when the train passes by and the type of underlying medium (ballast or slab track). This paper describes the selection and implementation stages of the system development that we propose and shares the test experiences obtained.

## 3. System Description

This study takes place as part of a whole system which includes a communication system from the sensor platform to a server application in a remote place. The communication system includes an installation application to configure the nodes in the slab installation phase. In both the slab installation and maintenance monitoring phases, the communication module is Digi’s Xbee PRO S5 with a frequency of 868 MHz. Symmetric encryption is used by this module to provide secure communications. It helps to improve the security of the system, but as future work it would be interesting to apply the model proposed in [19] to analyze the security, privacy and dependability of the whole system. 

**Figure 1:**
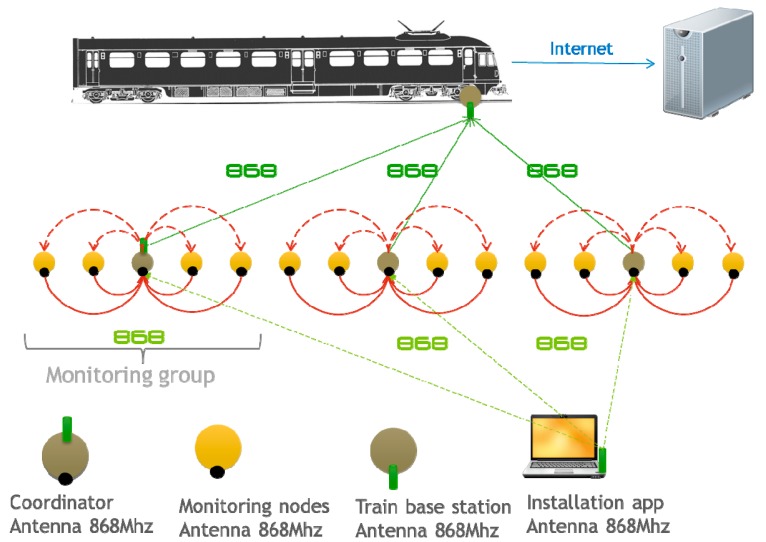
Sensor4PRI architecture.

Nodes are deployed inside a custom case. This custom case is inserted in the slab track inside a special hole carved into the concrete. This case is sealed and protected from unauthorized attacks and also from external weather conditions. In general terms, this casing approach allows operators to easily swap the existing case with a new one (with a node with similar configuration and a new battery inside). The sensors deployed in the system are used to obtain acceleration, inclination and distance information. They are divided into groups controlled by a cluster head (coordinator) that reports the information to base stations located in the passing trains. As described in Figure 1, the coordinator receives the information from the nodes of its group and establishes the communication with the train. The information obtained from the system can be used to assist operators in the installation phase of the slab tracks and also to assess the structural health of the railway infrastructure. By analyzing vibration performance (in the frequency domain), inclination and distance, abnormal situations can be detected and the evolution of potential defects controlled. Based on this railway application, a description of the node requirements is given in the next subsection.

In the wireless sensor network field it is well known that wireless transmission is one of the aspects that affect the life of the batteries the most, for this reason, it is worth remarking that the system is designed to retransmit as little information as possible in order to extend the life of the devices in terms of energy consumption. To address this issue nodes are organized in clusters which are controlled by a cluster-head. Furthermore, nodes are placed so that all nodes within the same cluster can communicate with their cluster-head in one-hop. Thanks to this design it is not necessary to carry out data fusion or aggregation techniques. Basically, the cluster-head receives information from its neighbors and retransmits it to one of the trains when it passes by. In the case the cluster-head is unable to retransmit all the information it will use the trains behind to complete its task. The cluster-head will always be able to transmit all the information received from their neighbors to the trains as they only generate data once a day (around 24 bytes) and furthermore, many trains pass by in a day, thus, a cluster-head has several chances to retransmit all the information.

This architecture allows the complexity of the applications to be reduced as nodes don’t need to transmit packets in a multi-hop way. The application of our system can be described in four simple actions:

1. Nodes read data from their sensors once a day.
2. Nodes send the information obtained from the sensor to their cluster-head.
3. Cluster-head sends the information received from the sensors to the train passing by.

In order to clarify the design of the on-board components and the communication mechanisms of the proposed platform, the set of requirements are detailed in this section. The integrated sensors have two main functions:

- Provide assistance in the installation phase of the slabs. The platform should provide an accurate estimation of the slab tilt and the distance between contiguous slabs. A graphical tool will analyze, in real time, the 3D position and distance to adjacent slabs.
- Provide information on structural integrity of the slabs and possible displacement (inclination and distance), vibration, etc. during their use. As for the previous objective, a tool to store and analyze information and gather real-time information will be developed.

During the installation phase, sensors integrated in board will have to give the information necessary to:

- Calculate the distance between slabs. This distance will not exceed 1 cm. with a tolerance of ±0.1 cm.
- Calculate the slope of the slab. It is necessary to achieve a precision of 0.17% (0.1 °C).

During the maintenance phase, the sensors will provide the information necessary to ensure the real-time knowledge of the state of the infrastructure:

- Acceleration readings of the slab tracks when trains are passing over them. In order to detect defects or abnormal situations, the frequency of the different vibrations obtained from the acceleration data is studied. To do this the raw acceleration data are transformed to the frequency domain by means of the Fast Fourier Transform (FFT). Due to the vibrating nature of slab tracks, acceleration must be obtained with a 0.005 g resolution, accuracy of 15% and bandwidth of 1 Hz–800 Hz.
- Displacement of the slab relative to its original position recorded during the installation phase.
- Alarms for low battery and sensor failure.

The goal of the system is to improve the safety of the infrastructure by allowing early detection of defects, even before they are visually noticeable. This will reduce maintenance interventions and costs as long as the lifespan of the system is reasonably high. In order to support this, the following systems of energy production will be studied for recharging the batteries:

- Vibrations generated by the train captured by piezoelectric sensors.
- Solar panels
- Thermal gradient

Finally, the integration mechanism of the encapsulation in the slab should allow extraction so that maintenance tasks (replacement of faulty sensors, battery change, *etc.*) can be easily carried out. The node must be accessible and will provide a plug & play attachment.

**Figure 2:**
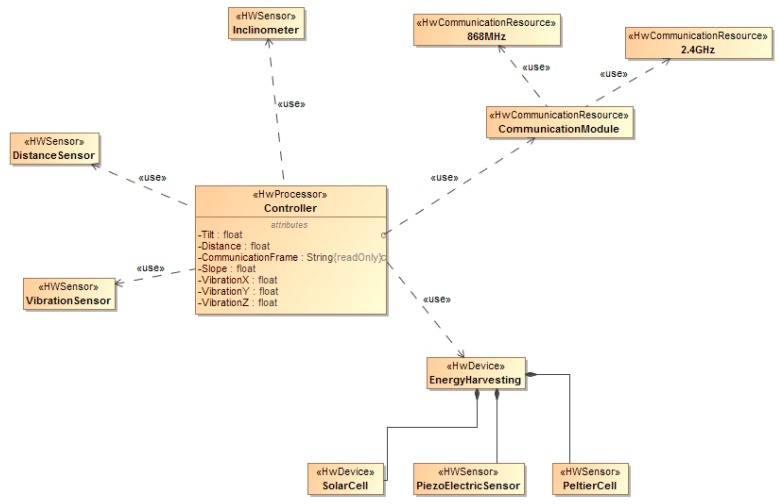
Hardware Architecture.

In Figure 2 the high level architecture for the hardware components of the system is shown. The system is composed of a microcontroller that controls different sensors. The system is powered by a battery and optionally by an energy harvesting module. The module is installed inside selected slab tracks to monitor different parameters as described in this section.

## 4. Platform and Sensors Implementation and Evaluation

In order to design the sensor platform, a set of different experiments with different hardware components has been carried out. This section describes the tests and their results. The following subsections detail the selected sensor platform (Section 4.1) and the sensor tests that have been carried out (Section 4.2).

### 4.1. Sensor Platform 

All reviewed hardware platforms are listed in Table 1. Regarding the two Arduinos, the MEGA 2560 model is a better quality model, bigger, with better technical characteristics and a higher cost (MEGA: 41 €; UNO: 20 €). A key difference is that Arduino UNO has only one serial port, while the Arduino MEGA has four, making it more flexible and useful. Additionally, the Arduino Mega has four times more SRAM which is very important to allow implementing some algorithms which are important for the proposed system and are described in the following sections (e.g., FFT analysis). On the other hand, BeagleBone Black and Raspberry PI are less constrained devices but with higher energy consumption. The most viable option both from an economic point of view and for flexibility reasons is the use of the open code platform Arduino. This platform offers a good balance between capabilities and energy consumption at a reasonable price. The MEGA 2560 model has been chosen as it provides more than one serial port which is necessary for some of the sensors selected in the following sections.

**Table 1:** Technical characteristics of the sensor boards evaluated.

**Name**	Arduino UNO	Arduino MEGA 2560	BeagleBone Black	Raspberry PI
**Microcontroller**	ATmega328	ATmega2560	AM335x ARM Cortex-A8	ARM1176JZF-S
**Operating Voltage**	5 V	5 V	5 V	5 V
**I/O Pins**	14 (6 PWM output) + 6	54 (14 PWM output) + 16	2x 46 pin headers	25 pin headers
**Flash Memory**	32 KB (0.5 KB bootloader)	256 KB (8 KB bootloader)	4 GB	SD dependant
**SRAM**	2 KB	8 KB	512 MB DDR3	256 MB
**EEPROM**	1 KB	4 KB	-	-
**Serial (UART)**	1	4	4	1
**Clock Speed**	16 MHz	16 MHz	1 GHz	700 MHz
**Price**	20 €	41 €	42 €	28 € + sd

We note that although a prototyping platform like Arduino has been chosen for the prototype, the final release of the product will more than likely use a custom-made sensor board tailored to the project requirements which means that energy consumption and costs can be considerably reduced.

### 4.2. Sensors

#### 4.2.1. Accelerometers

In Sensor4PRI the accelerometer is the main source of information for evaluating the structural health of the railway infrastructure. On the one hand, a precise accelerometer is essential for detecting subtle changes in the vibration patterns of the slab track. On the other hand, an economically viable accelerometer is needed to keep the cost of the monitoring system low. In order to evaluate these two factors a test has been carried out with the sensors whose main features are shown in Table 2. 

**Table 2:** Technical characteristics of evaluated sensors

	ADXL345	SQ-SVS
**Manufacturer**	Analog Devices	Signal Quest
**Axis**	3	2
**Consumption**	20 μA	40–145 μA
**Price**	21 €	363 €
**Interface**	SPI, I2C	Analog, UART
**Precision**	2 g–16 g	2 g
**Image**	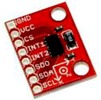	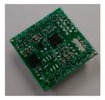

As these two accelerometers differ greatly in price, the goal of this test is to confirm that both sensors are able to detect vibration patterns in a similar way. If that is the case, then the ADXL345 sensor is going to be selected as it has a lower price.

**Figure 3:**
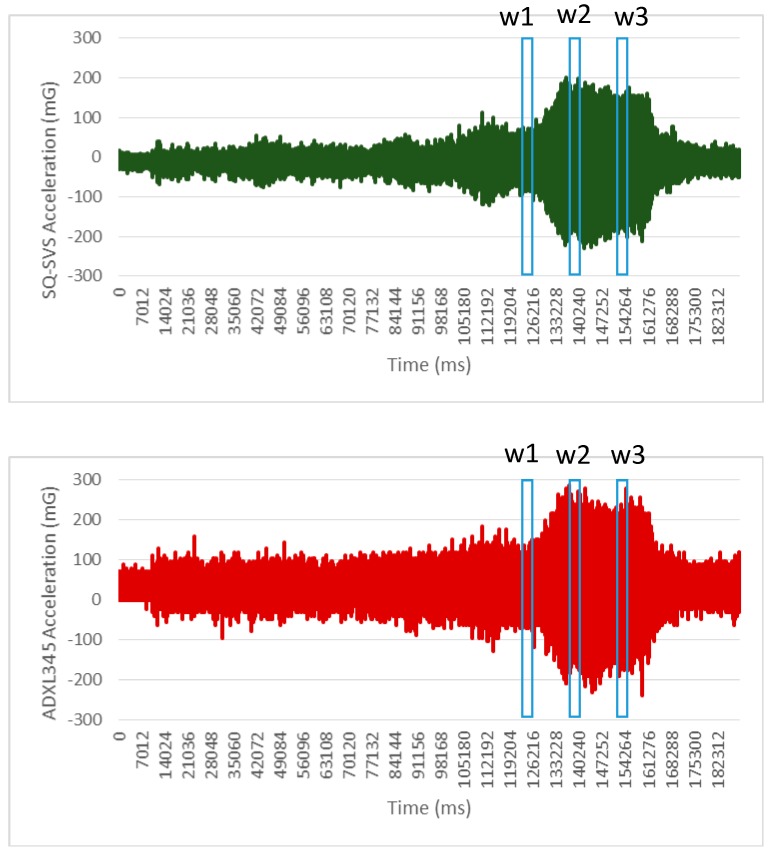
Raw acceleration (axis x) obtained for the sensors SQ-SVS and ADXL345.

The tests have been performed by attaching both sensors to a rotating electric motor. Both sensors are controlled by the Sensor4PRI system and collect raw data about acceleration simultaneously at a rate of 250 Hz. The total duration of the test is 3 min. Two/three axis data have been collected for SQ-SVS/ADXL345 sensors respectively although only results from axis X are presented in Figure 3 due to space reasons.

Figure 3 shows that although there are some differences in acceleration data perceived by these two sensors it is difficult to assess the performance of these two accelerometers using only this information.

**Figure 4:**
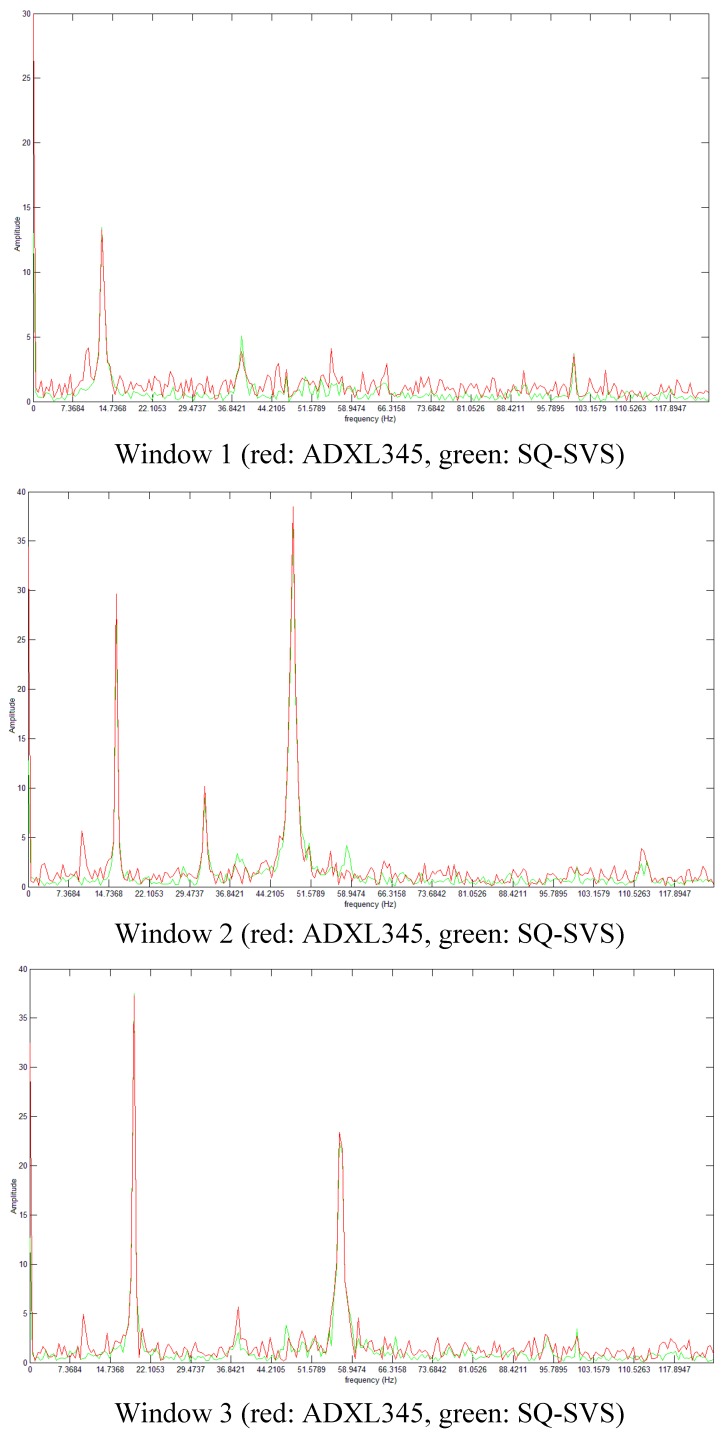
Frequency domain analysis of the raw data obtained in the test.

In the study of structural health of infrastructures, one of the most common techniques is to analyze the frequency of the different components of the acceleration signal. This information is used to detect changes in vibration patterns and abnormal acceleration response of the infrastructure that evidence the existence of a defect. The raw data acceleration obtained in the test has been transformed to the frequency domain by means of a FFT algorithm. This transformation is the one that the Sensor4PRI system applies to the data it collects. Figure 4 shows the result of this analysis for three different time windows of size 512. These three selected windows are marked in Figure 3 as w1, w2 and w3 respectively. The results show that peaks in frequency vibration are successfully detected in the same way by these two sensors. Therefore, this analysis confirms that both sensors detect the same vibration pattern in the electric motor. The same behavior is also obtained from these two sensors for the rest of windows in the test.

The results obtained in the test show that the use of a low cost accelerometer to obtained acceleration information in the frequency domain in this application domain seems feasible. As a result we have selected the ADXL345 sensor for installation in Sensor4PRI.

#### 4.2.2. Inclinometers

The system has to measure the tilt of the slabs in the installation phase to assist operators and to periodically monitor the slab behavior in the monitoring phase. Three alternatives have been considered to sense the slab tilt. Table 3 shows the technical characteristics of the evaluated inclinometers.

**Table 3:** Technical characteristics of the evaluated inclinometers.

	SQ-SI-360DA	SCA100T-D2	ADXL345
**Manufacturer**	SignalQuest	DFRobot	Analog devices
**Axis**	2	2	3
**Consumption**	4.6 mA	4 mA	40 μA
**Price**	363 €	63.14 €	16 €
**Interface**	analog, UART	SPI	SPI
**Precision**	Analog: 3.49% (2°) Digital: 0.17% (0.1°)	0.01% (0.0035°)	0.17% (0.1°)
**Image**	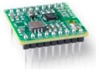	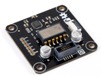	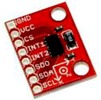

The first one is using the ADXL345 accelerometer described in Section 4.2.1. In this case, it is necessary to implement some simple transformations to get tilt results from acceleration data provided by the accelerometer. This solution is cheap and implies low energy consumption. However, as established in the system requirements, it is necessary to achieve a precision of 0.1 grades, which is not supported by this sensor. The second option is the use of the SQ-SI-360DA inclinometer from SignalQuest. This sensor automatically calculates the tilt from multiple acceleration readings carried out transparently with respect to the application. 

As a third option the SCA100T-D2 sensor was also analyzed. This sensor offers practically the same features as the SQ-SI-360DA sensor, so it not only meets with the project requirements, but it is also cheaper. To be sure that the performance of the SCA100T-D2 is similar to its competitor in terms of project requirements, both of them were tested in our lab in order to see the angle provided by them placing them in different positions and also change from one position to another gradually. The results show that SCA100T-D2 provides the same accuracy measures as the SQ-SI360DA sensor. Taking into account the tests and the kind of interface they offer, the SCA100T-D2 sensor is the best option.

#### 4.2.3. Distance Sensors

In order to measure the distance between slab tracks it is necessary to allocate one or two distance sensors in the adjacent borders of two slab tracks. On the market there are two well-known kinds of distance sensors: infrared and ultrasonic. The former are recommended for use principally in indoor environments since their measurements are highly affected by sunlight. That is, since light does not reflect the same way off every surface, the infrared sensor reading will be different for different surfaces, different colors, and different shades even if the range is the same. However, ultrasonic sensors not only can be used outside in bright sunlight but also they are able to carry out accurate measurements in these conditions. Due to the fact that the designed monitoring device is going to be installed outdoors, three ultrasonic distance sensors have been analyzed in order to choose the most suitable one: MB1043, SRF08 and PING ultrasonic sensor. Table 4 shows their main features.

**Table 4:** Technical features of the evaluated distance sensors.

	MB1043	SRF08	PING
**Manufacturer**	MaxBotix	Devantech	Parallax
**Consumption**	3.1 mA	3 mA	35 mA
**Price**	28 €	42 €	24 €
**Interface**	Analog, UART	Digital	Analog
**Image**	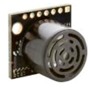	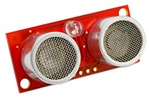	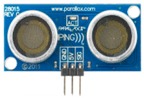

**Figure 5:**
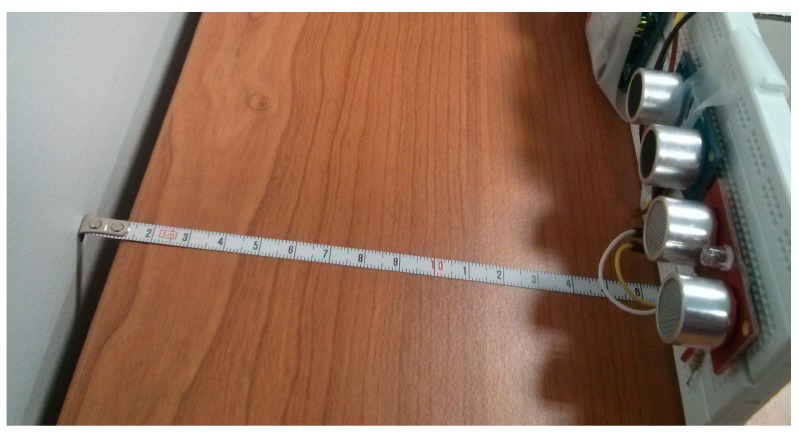
Short distance test of the ultrasonic distance sensors.

After testing in our lab (see Figure 5) the performance of the sensors presented in the Table 4, we realized that sensors with a dual-element ultrasonic module are able to read much closer distances than those with a single-element. Namely, the MB1043 sensor was able to read distances of between 30 cm and 5 m, the SRF08 sensor distances between 3 cm and 6 m, and the PING sensor distances between 2.41 cm and 3 m. Over short distances, the PING model was the sensor able to provide better and more accurate measurements. However, SRF08 was the model able to measure longer distances (up to 11 m) although when we tried to measure distances of 2.5 m, again, the PING sensor provided more accurate results. It is worth noting that both sensors were able to detect changes with a precision of 1–2 mm. In conclusion, we find that the SRF08 model is a highly configurable sensor. For instance, it can be tuned to performance faster reads over short distances, its gain can be modified and it can be also put into Artificial Neural Network mode to detect several obstacles located at different distances at the same time. But, from the project requirements point of view the most important aspects to be taken into account are energy consumption and accuracy measurements over short distances. Thus, despite the fact that the SRF08 sensor consumes much less energy than the PING sensor, we consider the latter to be more suitable for this project as it is not only able to provide accurate measurements when the obstacle is located very close but it is also half the price.

We are aware that the requirements state that the minimum measurable distance in this sensor is approximately 1 cm while the distance between slab tracks could be lower, but this issue can be solved by installing the sensor and setting it back from the slab track edge.

### 4.3. Railway Standard

The work presented here is part of a larger project in which several requirements have been defined which must by complied with. Among them there are two CENELEC standards which must be taken into account. They are:

- CENELEC 50121: Electromagnetic compatibility.
- CENELEC 50128: Rail applications—Communications, signaling and processing systems—Safety related electronic systems for signaling.

## 5. Energy Harvesting Evaluation

In order to evaluate the energy consumption of the monitoring system it is essential to determine the consumption of each separate system component. Table 5 shows the consumption measured in various operating modes of an Arduino node using the class library JeeLib for the “deep sleep” mode and an Xbee 868 Mhz radio transmitter.

**Table 5:** Energy consumption in different operating modes.

Operating Mode		Consumption (mA)
Transmitting maximum power (400 mW)	Awake mode Radio transmitter on Sensor off	(150–750 mA)
Transmitting (300 mW)	Awake mode Radio transmitter on Sensor off	150–270 mA
Receiving	Awake mode Radio transmitter on Sensor off	114 mA
Sensing	Awake mode Radio transmitter off Sensor on	53 mA
Sleeping	“Deep sleep” mode Radio transmitter off Sensor off	39 mA

Some conclusions can be drawn from the results. On the one hand we see that the major source of consumption is the radio transmitter, especially when it is transmitting data. We can obtain a significant energy saving if the transmission power is not too high, but this reduces the transmission range. On the other hand, the consumption when the radio is turned on but not transmitting (only receiving) is not negligible and therefore it is essential to turn the radio off when it is not in use.

Regarding the data gathering process, we have observed that the ADXL345 sensor has an almost negligible consumption (in the order of uA). In the “sensing” mode most of the consumption comes from the Arduino board itself, not from the sensor.

Finally, and contrary to what was expected, in the “deep sleep” mode the energy consumption of Arduino is relatively high, although the microprocessor is off. This is due to the voltage regulator incorporated inside the Arduino board. This regulator is responsible for transforming the voltage input (7–12 V) to the voltage actually needed by the board (5 V). This voltage regulator runs constantly even when the processor is off and produces high power consumption. Bearing in mind that the board will be in the “deep sleep” mode most of the time, it is essential to optimize the energy consumption in this mode. A possible solution to this problem would be to cancel the voltage regulator in the Arduino board and to manage this issue through an external device or battery more efficiently.

Anyway, due to the high energy consumption of the board, some mechanisms are needed to increase the life time of the battery that supplies energy to the board. For this reason, different energy harvesting devices were selected to determine the best suited to our requirements. The following subsections discuss them in the context of railway infrastructures.

### 5.1. Solar Panel

Solar panels are highly suitable for outdoor systems. However, slab tracks can be installed in indoor environments, as for example, tunnels. Both situations were studied to see whether the panels can be installed in the whole track or whether it was necessary to integrate different energy harvesting systems. Table 6 and Table 7 show the technical characteristics of the solar panel and the battery used to perform our tests, respectively. 

**Table 6:** Technical characteristics of the solar panel used.

	1.5 W Solar Panel 81 × 137
**Supplier**	Cooking hacks
**Type**	Cristal
**Power**	1.5 W
**Voltage**	5 V (peaks up to 10 V)
**Efficiency**	17%
**Price**	6.65 €
**Size**	81 × 137 × 2 mm
**Connector**	JST PH-2
**Image**	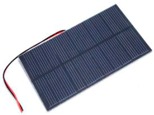

**Table 7:** Technical characteristics of the battery used.

	063450
**Manufacturer**	Unionfortune Electronic
**Voltage**	3.7 V
**Charge **	1000 mAh
**Price**	9.1 €
**Size**	53 × 33 × 5.7 mm
**Connector**	JST PH-2
**Image**	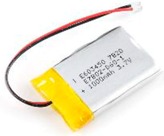

**Figure 6:**
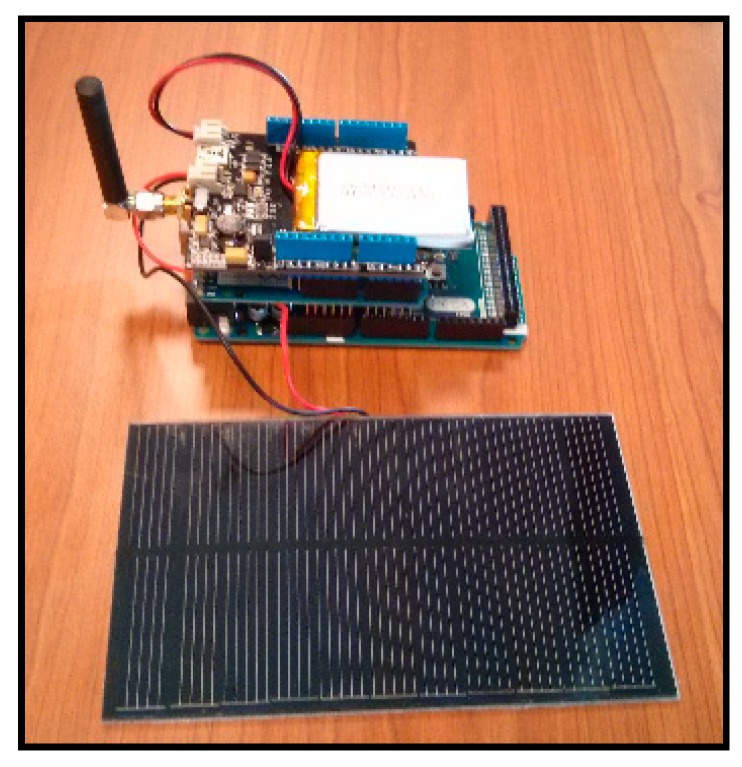
The whole system used to carry out solar panel tests.

The whole system can be seen in Figure 6. A shield Xbee Arduino containing Xbee PRO 868 and an Arduino Mega 2560 board module has been used. The Xbee module is used to send the results of voltage from the board to a PC where the data is collected and stored. Moreover, a special board responsible for efficiently transferring energy captured by the solar panel to the battery is also needed.

To study the impact of solar panels on the energy consumption issue, three tests were performed:

- Test 1. It does not use solar panels at all, in order to estimate the duration of the battery without using energy harvesting mechanisms. In this test, the device sends 100 byte packets for 10 s. After that, the node changes to the reception mode for 60 s and it returns again to the transmission mode and so on to drain the battery. This test simulates the situation where the device detects the train and begins transmitting data for 10 s and switches to the reception mode to collect data captured by the neighbor nodes.
- Test 2. Like the previous situation but connecting the solar panel.
- Test 3. It also uses the solar panel, but in this case it simulates the real behavior of the monitoring node (scheduled to the train timetable). This node wakes up once every hour, reads the accelerometer data and sends packets for 10 s. Then, the node changes to sleep mode for one hour, and so on.

**Figure 7:**
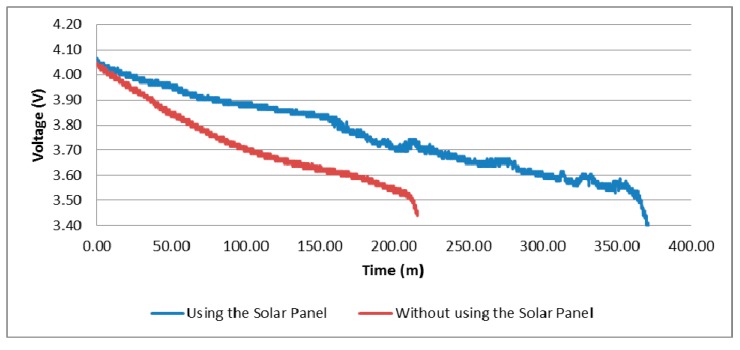
Battery draining in test 1 and 2.

Figure 7 shows the results of tests 1 and 2. When the solar panel is used, the battery lasts 164 min more and is able to transmit 10.952 additional packets.

**Figure 8:**
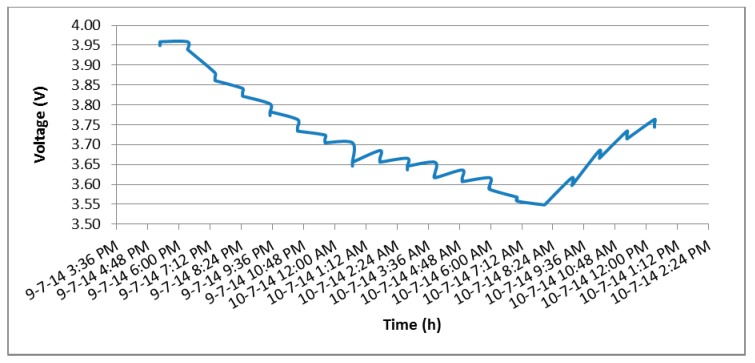
Battery charging/discharging in test 3.

Figure 8 shows how the solar panel is able to generate enough power to keep the node indefinitely on. Throughout the day the battery enters a discharging (9/7/14 6:00 p.m.) or charging (10/7/14 8:24 a.m.) phase depending on the way the sun’s rays affect the solar panel. In both phases different stepped zones can be appreciated. These correspond to small voltage drops which occur every hour during transmission mode.

### 5.2. Piezoelectric

A piezoelectric device can be used in our platform to obtain energy from the vibrations produced when trains pass over the slab track. Two different models have been analyzed. Table 8 shows their technical features. Additionally, a Mide EHE004 AC to DC converter and a 200 μF capacitor have also been used. The EHE004 converter can have two configurations: SS, recommended for low amplitudes, and PN for high amplitudes.

**Table 8:** Technical characteristics of V20W and V21B piezoelectric devices from Mide.

	V20W	V21B
**Frecuency ranges**	75 Hz–175 Hz	80 Hz–205 Hz
**Performance data**	180 Hz, 1 g, 1.719 mW 130 Hz, 1 g, 2.692 mW 95 Hz, 1 g, 3.005 mW 75 Hz, 1 g, 5.860 mW	275 Hz, 1 g, 0.250 mW 175 Hz, 1 g, 0.658 mW 140 Hz, 1 g, 1.311 mW 105 Hz,1 g, 2.252 mW
**Price**	65.75 €	48.1 €
**Size**	81 × 33.3 × 0.86 mm	69 × 14.5 × 0.78 mm
**Image**	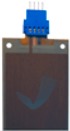	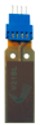

Table 9 shows the results of the different tests. All of them were conducted considering vibrations for 81 s, which is the estimated time of trains passing throughout the day.

**Table 9:** Piezoelectric tests.

Test	Box	Piezoelectric	EHE004 Configuration	Voltage	Coulombs (C)	mAh
1	Metal	V20W	PN	2.7 V	0.00054	0.00015
2	Metal	V20W	PN	5 V	0.001	0.00027
3	Metal	V20W	SS	7.7 V	0.00152	0.00042
4	Metal	V21B	SS	5.4 V	0.00108	0.00030
5	Plastic	V20W	SS	4.4 V	0.00088	0.00024

These results definitively show that the use of vibration piezoelectric devices to harvest energy is currently an unviable solution. There exist other piezoelectric systems able to generate energy from pressure [20]. This novel system is able to generate a great deal of energy (about 120 KWh per 1 km rail) but its design makes it really difficult to integrate into our monitoring device and its installation depends on the installation of the slab track.

### 5.3. Peltier Cell 

The main objective of using Peltier cells is to check whether or not they are capable of generating energy from temperature differences produced in the tunnels, as in these environments, solar panels cannot be used due to the total absence of light. The technical characteristics of the cell used in our tests are shown in Table 10.

**Table 10:** Technical characteristics of the Peltier cell used.

	PELTIER CELL TEC1-12710HTS 100 W 40 × 40 mm
**Power**	100 W
**Maximum Voltage**	15.4 V
**Connectors**	No (conventional positive and negative cables)
**Current**	10.5A
**Size**	40 × 40 × 3.3 mm
**Temperature range**	66 degrees Celsius
**Price**	22.74 €
**Image**	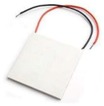

**Figure 9:**
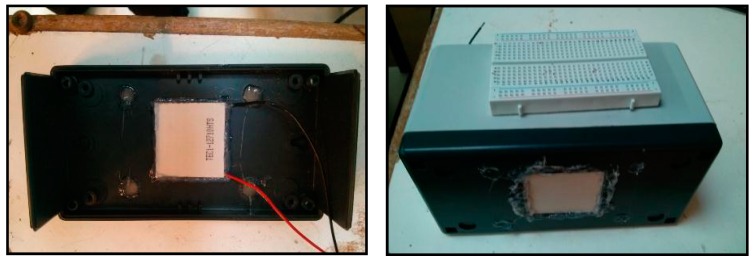
Peltier cell installation.

The test goal is to check whether the temperature difference established between the exterior of the platform casing in contact with the concrete of the slab track and the inside of the casing will be used by the Peltier cell to generate energy. The bottom of the casing was modified to embed the Peltier cell and the edges where it fits were sealed with silicone (Figure 9). This way we achieve stability in the thermal difference and a firm clamping of the cell. 

**Figure 10:**
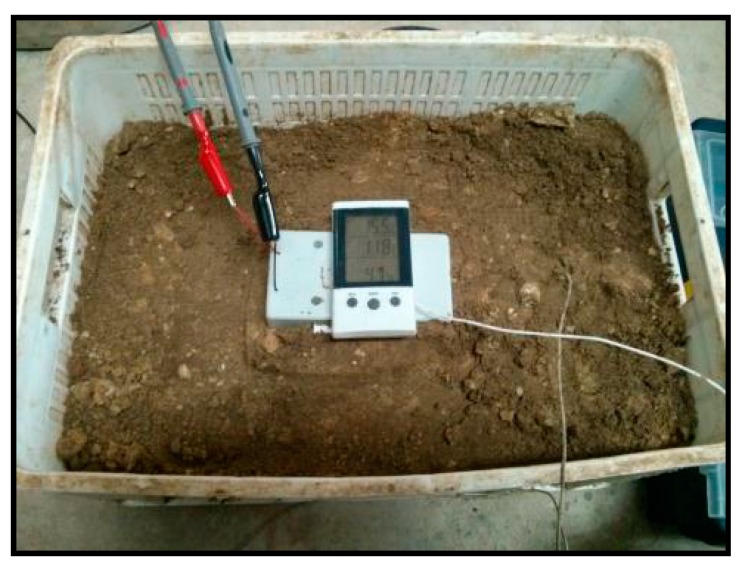
Peltier cell installed inside a plastic case and buried in the sand.

The environment of the slab track has been simulated using a box filled with sand on which the platform casing has been placed (Figure 10) so that the face of the Peltier cell was in contact with the sand.

Two experiments were defined:

*a)*  *To place the platform indoor, in a similar environment to railway tunnels.* 

**Figure 11:**
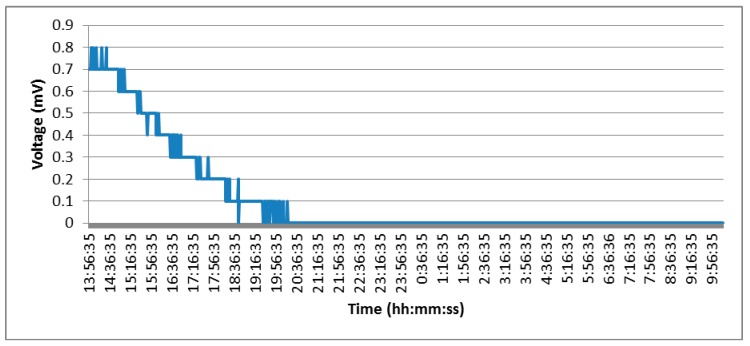
Energy generated by the Peltier cell inside a “tunnel”.

To simulate the environment of a railway tunnel, the sand box was placed in a basement where the temperature is low and stable, which is a serious drawback for the performance of this device. Figure 11 shows the voltage generated by the Peltier cell over a period of 20 h. After installation it was observed how a small voltage was generated probably due to the initial temperature of the sand, which was below room temperature. As time passes the temperature difference decreased until it was practically 0, hence the voltage generated was also 0.

*b)*  *To place the platform outside, to appreciate the possible obtained differences* 

**Figure 12:**
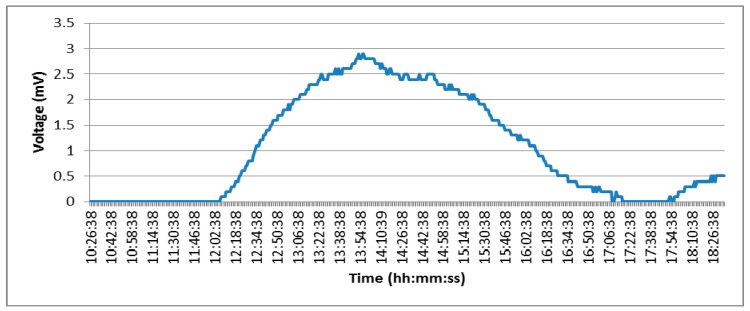
Energy generated by the Peltier cell outside a “tunnel”.

This test consisted of placing the sand box in a window of the room where it went through periods of direct light, shadow and absence of light. Each of these three phases is reflected in Figure 12. From 10:00 h to 12:08 h in the morning the box was in shade and therefore the temperature difference was 0 °C, hence the voltage generated was also 0. From 12:08 h to 14:00 h the sun’s rays begin to warm up the box and as the sand temperature was kept fresh a temperature gradient was produced and thus voltage was generated. After this period of time, the sun started to move away (from 14:00 to 17:15 h), and this caused the temperature of the interior of the platform case began to fall until it reached the same temperature as the sand. Finally there was another period (17:04 to 18:32) in which the Peltier cell began to generate power again. This is probably because the sand retained the temperature during the period of time it was exposed to the sun while the interior of the platform casing began to cool.

Our tests concluded, we can say that the Peltier cell is an option to consider for energy harvesting if they are going to be placed in areas that are exposed to the sun, but obviously in these areas solar panels give much better results. According to the results obtained in the first experiment (indoor installation) we do not recommend using Peltier cells in the tunnels since the thermal variations that occur are negligible. In this situation, it is necessary to have a battery with the necessary voltage to guarantee long duration.

## 6. Cost Analysis of the Sensor4PRI Platform

**Figure 13:**
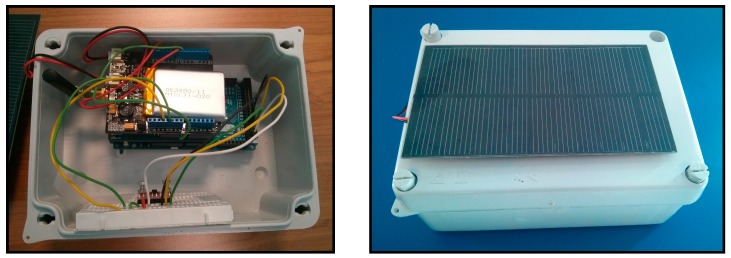
Sensor4PRI platform.

The previous sections have studied the main components that could be integrated in the system proposed in this paper. Taking into account their characteristics, the final prototype is composed by the components shown in Table 11. The components have been selected taking into account the accuracy of the sensors, the energy consumption and the reduced costs. The Sensor4PRI platform is pictured in Figure 13. 

It is important to highlight that the cost of the final monitoring device could be reduced a lot for two obvious reasons. First, because all the components can be integrated into a single electronic board and second, because the device is designed to be installed along the railway, which means that a high number of devices will be produced and the costs can be reduced.

**Table 11:** Selected components.

Component	Price
Arduino Mega	41.00 €
Module Xbee-Pro 868	75.00 €
Xbee explorer regulated	8.50 €
Antenna 0 dBi	8.00 €
Battery Litio 1000 mA/h–3.7 V	9.10 €
Shield solar cell	11.50 €
Solar Panel 1.5 W	6.65 €
Acelerometer ADXL345	21.00 €
Inclinometer	63.14 €
Distance Sensor SRF05	16.00 €
Open Log	24.95 €
SD card	6.00 €
Total	290.84 €

## 7. Conclusions and Future Work

In this paper, a sensor platform called Sensor4PRI has been presented to provide a system to cost-effectively monitor railway infrastructures using Wireless Sensor Networks. The technical features of the different platform components have been shown, together with the exhaustive experiments carried out in order to analyze and evaluate them.

The sensor platform has been specifically designed be part of a slab track, to be inserted in it during its construction, so that it can be used in both installation and maintenance phases of these kinds of systems. Distance sensors and inclinometers are used in the installation phase helping operators to place the slabs in the right position. Accelerometers are in charge of collecting information on vibrations suffered by the infrastructure when a train travels over it, achieving the supervision and maintenance of the infrastructure’s structural health. In addition, solar panels have been included to increase the life time of the battery that supplies energy to the board.

As for future work, we are involved in the deployment of several Sensor4PRI platforms along a section of railway infrastructure (we still need to get the corresponding permission from the authorities) and, using the data mule based communication mechanism described and simulated in our previous approach, we hope to achieve a real WSN-based monitoring system for railway infrastructure protection.
